# Current Management of Uncomplicated Acute Appendicitis: A Narrative Review of Nonoperative and Surgical Strategies

**DOI:** 10.7759/cureus.106086

**Published:** 2026-03-29

**Authors:** Sol Angie Rincon Mora

**Affiliations:** 1 Surgery, Sanitas University Foundation, Bogota, COL

**Keywords:** acute appendicitis, antibiotic therapy, appendectomy, laparoscopic appendectomy, nonoperative management

## Abstract

Acute appendicitis remains one of the most common surgical emergencies worldwide and has traditionally been treated with appendectomy. However, growing evidence from randomized trials and international guidelines has challenged this paradigm, suggesting that nonoperative management with antibiotics may be a safe alternative for selected patients with uncomplicated disease. This narrative review aims to examine the current evidence on the management of uncomplicated acute appendicitis, with particular focus on the role of antibiotic therapy compared with surgical appendectomy, patient selection, and clinical outcomes. A narrative review of the literature was conducted using major medical databases, including PubMed, Scopus, and Google Scholar. Randomized clinical trials, systematic reviews, meta-analyses, and international guidelines published in recent years were analyzed to summarize current diagnostic strategies and treatment approaches for uncomplicated acute appendicitis. Recent studies demonstrate that antibiotic therapy can successfully treat a substantial proportion of patients with uncomplicated appendicitis, potentially avoiding immediate surgery. Nevertheless, nonoperative management is associated with higher rates of recurrence and subsequent appendectomy. The presence of an appendicolith has been consistently identified as a significant predictor of treatment failure. Laparoscopic appendectomy remains a safe and definitive treatment with low complication rates and minimal risk of recurrence. The management of uncomplicated acute appendicitis is evolving from a strictly surgical disease to a condition with multiple evidence-based treatment options. While appendectomy remains the definitive therapy, antibiotic treatment represents a feasible alternative in carefully selected patients. A patient-centered approach that incorporates imaging findings, clinical risk factors, and shared decision-making is essential for optimal outcomes.

## Introduction and background

Acute appendicitis remains one of the most common surgical emergencies worldwide and a frequent cause of acute abdominal pain requiring urgent evaluation. It continues to represent a significant burden on healthcare systems, and its diagnosis and management remain highly relevant in contemporary surgical practice [1]. Traditionally, appendectomy has been considered the standard treatment because it provides definitive source control and virtually eliminates the risk of recurrence [2].

Advances in diagnostic imaging and minimally invasive surgery have significantly influenced management strategies. Accurate differentiation between uncomplicated and complicated appendicitis has become essential, as treatment approaches and outcomes differ considerably between these entities [3]. In this context, increasing interest has emerged in the nonoperative management of selected patients with uncomplicated appendicitis [4].

Randomized clinical trials have shown that antibiotic therapy can successfully treat a substantial proportion of patients, allowing some to avoid surgery during the initial episode [5]. However, nonoperative management is associated with recurrence and the potential need for delayed appendectomy [6]. Comparative studies further suggest that both surgical and antibiotic-based approaches may be reasonable in selected patients, each with distinct risk-benefit profiles [7]. Meta-analyses have reinforced that nonoperative management is feasible but not universally successful, highlighting the importance of appropriate patient selection [8].

Given the evolving evidence and the growing emphasis on individualized care, a contemporary review of the management of uncomplicated acute appendicitis is warranted. This narrative review aims to summarize current evidence on diagnostic evaluation, antibiotic therapy, appendectomy, patient selection, and clinical outcomes.

## Review

Methods 

A narrative review of the literature was conducted to summarize current evidence regarding the diagnosis and management of uncomplicated acute appendicitis. Electronic database searches were performed in PubMed/MEDLINE, Scopus, and Google Scholar for articles published between January 2000 and March 2026.

Search terms included combinations of the following keywords using Boolean operators (AND, OR): “acute appendicitis,” “uncomplicated appendicitis,” “nonoperative management,” “antibiotic therapy,” “appendectomy,” “appendicolith,” and “laparoscopic appendectomy.”

Randomized controlled trials, systematic reviews, meta-analyses, observational studies, and international clinical guidelines related to the diagnosis and treatment of uncomplicated acute appendicitis were considered eligible for inclusion. Priority was given to high-quality evidence, including randomized clinical trials and consensus guidelines from surgical societies.

Studies focusing exclusively on complicated appendicitis (e.g., perforation, abscess, or diffuse peritonitis), non-English publications, and case reports were excluded. Titles and abstracts were initially screened to identify potentially relevant studies, followed by full-text evaluation of selected articles.

The final selection prioritized studies with the highest methodological quality and clinical relevance. Methodological quality was assessed based on study design and relevance, giving preference to large randomized controlled trials, multicenter studies, systematic reviews, and meta-analyses, and international clinical guidelines from recognized surgical societies. Although a formal quality scoring system was not applied, studies were selected based on their methodological rigor, sample size, consistency of findings, and impact on current clinical practice.

Particular emphasis was placed on major randomized clinical trials comparing antibiotic therapy with appendectomy, including the appendicitis acuta (APPAC) trial and the comparison of outcomes of antibiotic drugs and appendectomy (CODA) trial.

As this study represents a narrative review of previously published literature, no primary patient data were collected, and therefore no sample size calculation or sampling strategy was required.

A total of 24 studies were ultimately included in this narrative review, comprising randomized clinical trials, systematic reviews and meta-analyses, and international clinical guidelines. Specifically, the review included six randomized clinical trials, eight systematic reviews and meta-analyses, and three guideline-based studies, all selected based on their methodological quality and relevance to the contemporary management of uncomplicated acute appendicitis.

Although a formal PRISMA framework was not applied, the study selection process followed a structured approach based on predefined inclusion and exclusion criteria, and search terms were adapted for each database to ensure comprehensive coverage of relevant literature.

Definition and diagnosis of uncomplicated acute appendicitis 

Uncomplicated acute appendicitis is generally defined as inflammation of the appendix without evidence of perforation, abscess, phlegmon, or diffuse peritonitis. This distinction between uncomplicated and complicated appendicitis is clinically important because it directly influences treatment decisions and patient outcomes [9]. Traditionally, the diagnosis of acute appendicitis was based primarily on clinical findings such as right lower quadrant abdominal pain, fever, leukocytosis, and localized peritoneal signs. However, clinical assessment alone may be insufficient, as the presentation of appendicitis can vary widely among patients [10].

**Figure 1:**
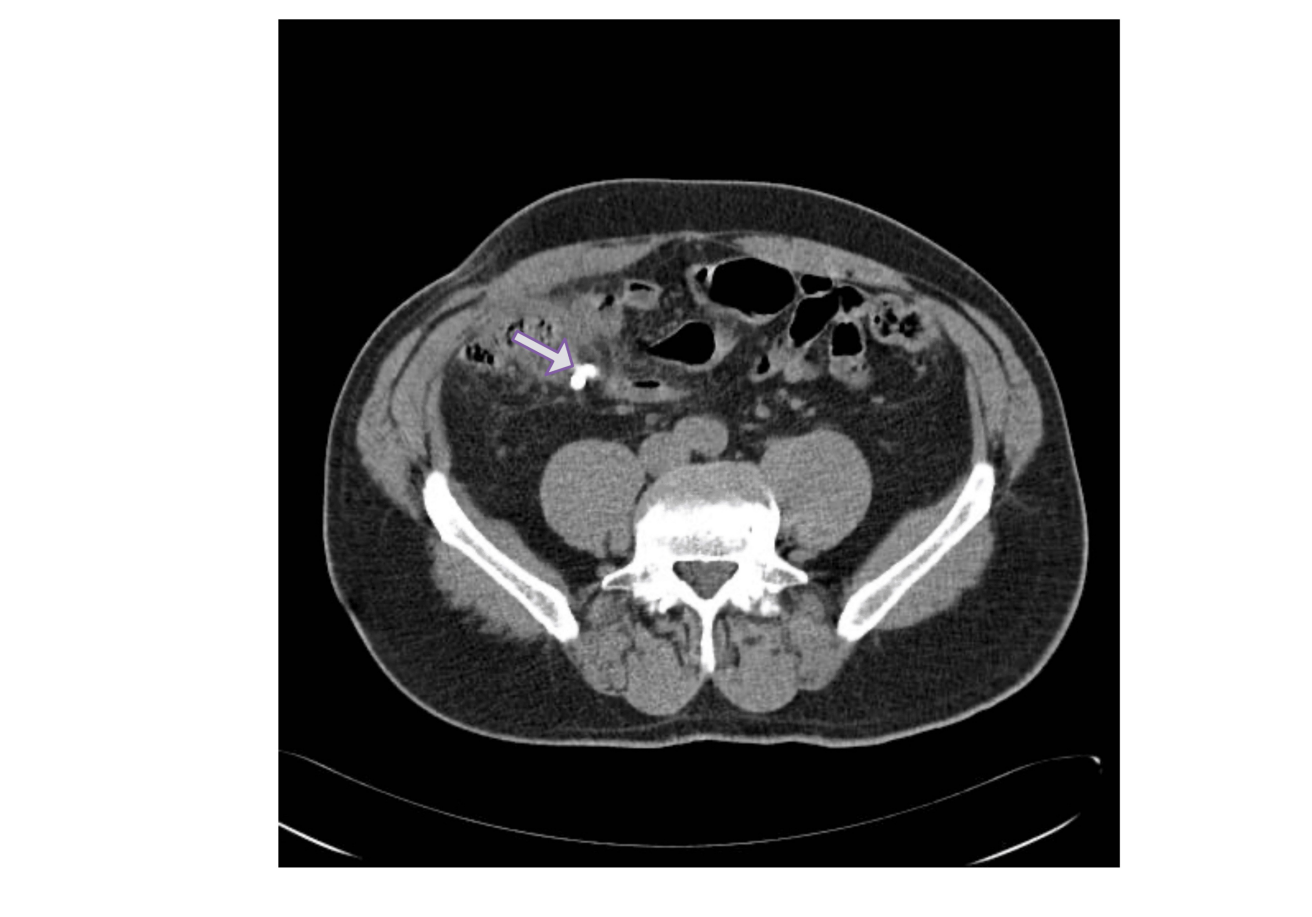
Computed tomography image demonstrating an inflamed appendix with an appendicolith (arrow) in the right lower quadrant Image adapted from Radiopaedia.org under Creative Commons Attribution-NonCommercial-ShareAlike license (CC BY-NC-SA 3.0) [11].

Advances in diagnostic imaging have significantly improved the accuracy of appendicitis diagnosis and the ability to distinguish uncomplicated from complicated disease. Computed tomography (CT) is currently considered the most accurate imaging modality for the diagnosis of acute appendicitis in adults, demonstrating high sensitivity and specificity. CT imaging also allows identification of important features such as appendiceal diameter, wall thickening, periappendiceal inflammation, and the presence of an appendicolith. An appendicolith appears as a hyperdense intraluminal calcification and has been associated with an increased risk of treatment failure in nonoperative management (Figure 1) [11,12].

Typical CT findings in uncomplicated appendicitis include an enlarged appendiceal diameter greater than 6 mm, appendiceal wall thickening, periappendiceal fat stranding, and luminal distension. Importantly, uncomplicated appendicitis is characterized by the absence of findings suggesting complicated disease, such as abscess formation, extraluminal air, phlegmon, or diffuse peritonitis.

Ultrasound is also widely used, particularly in younger patients and pregnant individuals, due to its lack of ionizing radiation. Although operator-dependent, ultrasound can demonstrate characteristic findings such as a non-compressible enlarged appendix and periappendiceal fluid [12]. In situations where ultrasound results are inconclusive, CT imaging is frequently used to confirm the diagnosis and evaluate disease severity.

Accurate classification of appendicitis has become increasingly important with the emergence of nonoperative management strategies. Imaging findings that suggest complicated appendicitis, such as abscess formation, extraluminal gas, or diffuse peritonitis, generally indicate the need for surgical management. In contrast, patients with imaging-confirmed uncomplicated appendicitis may be considered candidates for either appendectomy or antibiotic therapy, depending on clinical factors and patient preference [13].

Surgical management: appendectomy 

Appendectomy has long been considered the definitive treatment for acute appendicitis and remains the most commonly performed emergency surgical procedure worldwide. Surgical removal of the inflamed appendix provides immediate source control, eliminates the risk of recurrence, and has been associated with excellent clinical outcomes [14]. Historically, open appendectomy was the standard surgical approach; however, the widespread adoption of minimally invasive techniques has significantly changed contemporary surgical practice.

Laparoscopic appendectomy is currently the preferred surgical approach in most centers due to its well-established advantages over open surgery. Multiple studies have demonstrated that laparoscopic appendectomy is associated with reduced postoperative pain, shorter hospital stay, faster recovery, and earlier return to normal activities [15]. The typical laparoscopic technique involves a three-port approach, including an umbilical camera port, a suprapubic working port, and a left lower quadrant port.

Despite these advantages, both laparoscopic and open appendectomies remain safe and effective treatment options. Complication rates after appendectomy are generally low, particularly in cases of uncomplicated appendicitis. Reported complications may include surgical site infection, intra-abdominal abscess, and postoperative ileus, although their incidence remains relatively low with current surgical techniques and perioperative care [16].

Importantly, appendectomy offers a definitive solution for appendicitis, as recurrence does not occur following removal of the appendix. This characteristic represents one of the major advantages of surgical management when compared with nonoperative treatment strategies [17]. For this reason, appendectomy continues to be considered the gold standard therapy for many surgeons, particularly in patients with a low surgical risk.

However, the emergence of evidence supporting nonoperative management with antibiotics has introduced an important shift in the treatment paradigm. Increasing evidence suggests that selected patients with uncomplicated appendicitis may avoid surgery through antibiotic therapy, leading to ongoing debate regarding the optimal initial treatment strategy [18].

Nonoperative management with antibiotics 

Nonoperative management with antibiotics has emerged as a potential alternative to surgery for selected patients with uncomplicated acute appendicitis. This approach is based on the hypothesis that appendiceal inflammation in uncomplicated cases may be controlled with antimicrobial therapy, thereby avoiding immediate surgical intervention. Over the past two decades, several randomized clinical trials and observational studies have evaluated the effectiveness and safety of antibiotic therapy as the primary treatment for uncomplicated appendicitis [19-21].

One of the most influential studies in this field is the APPAC randomized clinical trial, which demonstrated that antibiotic therapy could successfully treat a substantial proportion of patients with uncomplicated appendicitis during the initial episode [4]. In this trial, approximately 70% of patients treated with antibiotics did not require appendectomy during the first year of follow-up. Long-term follow-up data from the same cohort reported a cumulative recurrence rate of approximately 39% at five years, indicating that although many patients initially avoid surgery, recurrence remains a relevant consideration [5].

Similarly, the CODA randomized clinical trial further contributed to the growing evidence supporting nonoperative management in selected patients [6]. This large randomized trial demonstrated that antibiotic therapy was noninferior to appendectomy in terms of short-term health status outcomes. However, a proportion of patients initially treated with antibiotics ultimately required appendectomy during follow-up [6,22].

Several randomized clinical trials have evaluated antibiotic therapy as an alternative to appendectomy for uncomplicated acute appendicitis. The main characteristics and outcomes of these studies are summarized in Table 1.

**Table 1:** Major randomized clinical trials evaluating antibiotic therapy versus appendectomy for uncomplicated acute appendicitis APPAC: appendicitis acuta, CODA: comparison of outcomes of antibiotic drugs and appendectomy.

Study	Year	Study design	Patients	Main findings
APPAC Trial: Salminen et al. [4]	2015	Randomized clinical trial	530	73% avoided surgery at 1 year
APPAC 5-year follow-up Trial: Salminen et al. [5]	2018	Follow-up study	530	39% recurrence at 5 years
CODA Trial: Flum et al. [6]	2020	Randomized clinical trial	1552	Antibiotics noninferior to surgery in short-term outcomes
Vons et al. [15]	2011	Randomized clinical trial	239	Higher recurrence with antibiotic treatment
Hansson et al. [16]	2009	Randomized clinical trial	369	Antibiotics effective in selected patients

Despite these promising results, nonoperative management is not without limitations. Several studies and meta-analyses have reported higher rates of recurrence, readmission, and delayed appendectomy in patients treated with antibiotics compared with those undergoing immediate surgical intervention [6]. Furthermore, certain radiologic findings, particularly the presence of an appendicolith, have been associated with an increased risk of treatment failure and complications in patients managed nonoperatively.

Consequently, antibiotic therapy is generally considered an appropriate option only for carefully selected patients with imaging-confirmed uncomplicated appendicitis who are clinically stable and able to undergo close follow-up. In such cases, shared decision-making between the patient and the healthcare team plays a crucial role in determining the most appropriate treatment strategy.

Predictors of treatment failure 

Although nonoperative management with antibiotics has demonstrated promising results in selected patients with uncomplicated acute appendicitis, treatment failure remains an important concern. Several clinical and radiological factors have been associated with an increased risk of recurrence, readmission, or the need for subsequent appendectomy in patients initially treated with antibiotics [14].

One of the most consistently reported predictors of treatment failure is the presence of an appendicolith. An appendicolith represents a calcified intraluminal deposit that may obstruct the appendiceal lumen. This obstruction increases intraluminal pressure, compromises mucosal blood flow, and promotes bacterial proliferation. As a result, inflammation may progress to gangrene or perforation even in patients receiving antibiotic therapy, which may explain the higher rates of treatment failure observed in patients with appendicoliths.

Multiple studies have demonstrated that patients with appendicolith have significantly higher rates of complications, recurrence, and eventual appendectomy when managed nonoperatively [5]. The presence of an appendicolith is thought to contribute to persistent luminal obstruction, which may promote ongoing inflammation and increase the likelihood of disease progression.

In addition to appendicolith, other factors may influence the success of nonoperative management. These include elevated inflammatory markers, larger appendiceal diameter on imaging, and extensive periappendiceal inflammatory changes. Some studies have suggested that patients with more severe inflammatory findings on computed tomography may be at greater risk of treatment failure when managed with antibiotics alone [6].

Patient-related factors may also play a role in treatment outcomes. For example, individuals with significant comorbidities, immunosuppression, or limited access to follow-up care may not be ideal candidates for nonoperative treatment. In such situations, surgical management may offer a more reliable and definitive approach [2].

**Figure 2:**
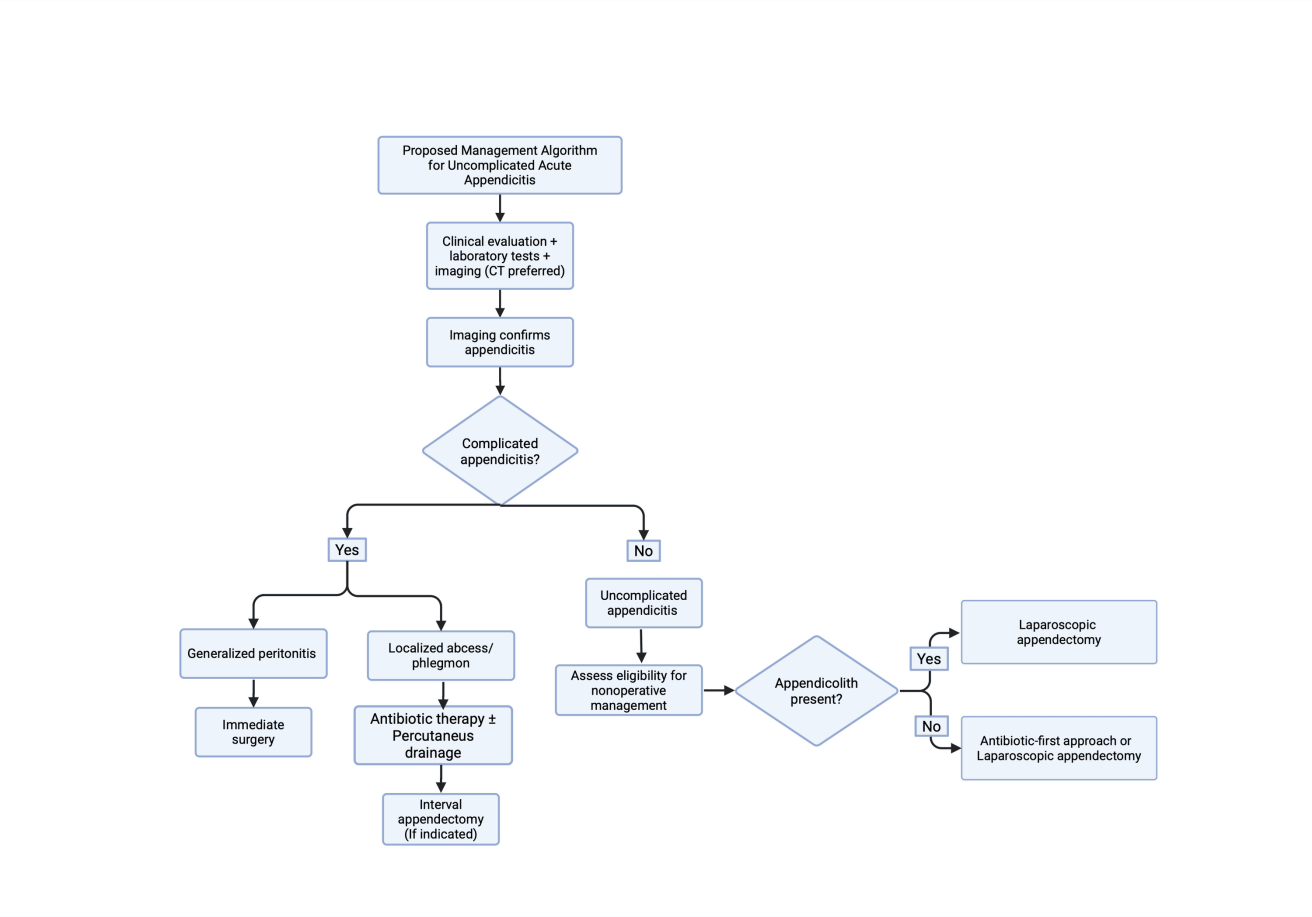
Algorithm for the management of uncomplicated acute appendicitis Proposed management algorithm for uncomplicated acute appendicitis. The algorithm distinguishes between generalized peritonitis and localized abscess/phlegmon in cases of complicated appendicitis. Created using BioRender.

Recognizing these predictors is essential for appropriate patient selection and for minimizing the risk of complications associated with delayed surgical intervention. Consequently, careful evaluation of clinical presentation, imaging findings, and patient-specific factors should guide the decision between operative and nonoperative management. The proposed management approach is summarized in Figure 2.

In cases of complicated appendicitis, management should be individualized. Patients with generalized peritonitis require urgent surgical intervention, whereas those with localized abscess or phlegmon may be initially managed with antibiotics and, when appropriate, percutaneous drainage, followed by interval appendectomy in selected cases. This approach is supported by current international guidelines [3,22]. This distinction is essential to provide a more evidence-based and contemporary management pathway.

Discussion 

The management of uncomplicated acute appendicitis has evolved considerably over the past decades. Several randomized clinical trials and observational studies have evaluated the effectiveness and safety of antibiotic therapy [20]. Historically, appendectomy has been considered the standard treatment, providing definitive resolution of the disease and eliminating the risk of recurrence. Advances in surgical techniques, particularly the widespread adoption of laparoscopic appendectomy, have further improved postoperative outcomes, including reduced postoperative pain, shorter hospital stays, and faster recovery times.

Nevertheless, growing evidence has challenged the concept that surgery is mandatory in all cases of uncomplicated appendicitis. Several randomized clinical trials evaluating antibiotic therapy versus appendectomy for uncomplicated appendicitis have been previously described (e.g., APPAC and CODA trials; Table 1) [6,20]. These studies suggest that antibiotic therapy may be a reasonable initial treatment option in selected patients, although it is associated with a higher risk of recurrence and subsequent appendectomy.

Similarly, the CODA trial further supported the role of antibiotics as an alternative initial treatment strategy [6]. Despite these encouraging results, nonoperative management has several limitations. Meta-analyses and systematic reviews consistently report higher rates of recurrence, treatment failure, and readmission among patients managed nonoperatively [20].

Patient selection, therefore, plays a crucial role when considering nonoperative management. The presence of an appendicolith has been associated with higher complication and recurrence rates in patients treated with antibiotics.

Current international guidelines emphasize that both appendectomy and antibiotic therapy may represent appropriate treatment strategies for selected patients with uncomplicated appendicitis [21,22]. Consequently, modern management increasingly relies on individualized decision-making based on imaging findings, clinical characteristics, and patient preferences.

This narrative review has several limitations. First, the narrative design may introduce selection bias because the included studies were not selected through a fully systematic methodology. Second, heterogeneity among studies regarding patient selection, antibiotic regimens, and follow-up duration may limit direct comparisons between trials. Finally, although randomized clinical trials provide important evidence, translating these results into diverse real-world populations may be challenging due to differences in healthcare systems, patient characteristics, and access to diagnostic imaging.

## Conclusions

The management of uncomplicated acute appendicitis has evolved considerably in recent years. While appendectomy remains the definitive treatment with a very low risk of recurrence, growing evidence supports nonoperative management with antibiotics as a feasible alternative in carefully selected patients. Randomized clinical trials and meta-analyses have demonstrated that antibiotic therapy can successfully treat a significant proportion of patients, although higher rates of recurrence and delayed appendectomy remain important considerations.

Consequently, the choice between surgical and nonoperative treatment should be individualized, taking into account clinical presentation, imaging findings, the presence of appendicolith, and patient preferences. Current international guidelines support a patient-centered approach that incorporates shared decision-making and careful risk stratification.

Future research should focus on refining patient selection criteria and identifying those most likely to benefit from nonoperative management, thereby optimizing clinical outcomes and resource utilization.
